# Pharmacogenomic Determinants of Interindividual Drug Response Variability: From Discovery to Implementation

**DOI:** 10.3390/genes12030393

**Published:** 2021-03-10

**Authors:** Stuart A. Scott, Jesse J. Swen

**Affiliations:** 1 Department of Pathology, Stanford University, Stanford, CA 94305, USA; 2 Clinical Genomics Laboratory, Stanford Health Care, Palo Alto, CA 94304, USA; 3 Department of Clinical Pharmacy & Toxicology, Leiden University Medical Center, 2333 ZA Leiden, The Netherlands

Since the term “pharmacogenetics” was first published in the late 1950s by Friedrich Vogel, the field has evolved into genome-wide association studies identifying novel variants associated with drug response phenotypes, international societies and consortia dedicated to pharmacogenomic research and clinical implementation, clinical practice guidelines, and the increasing availability of pharmacogenomic tests for healthcare providers in both hospital and primary care. However, current challenges related to translating pharmacogenomic discoveries into clinical practice include uncertain regulatory oversight, best practices for pharmacogenomic testing and interpretation, ongoing debate over clinical validity/utility, and clinical provider education and adoption. Moreover, the implementation of current pharmacogenomic knowledge introduces novel challenges for the field. How to manage the interplay between gene–drug and drug–drug interactions? How to deal with rare variants or variants of unknown significance? In this Special Issue, fifteen papers were published, which showcase novel and international research in pharmacogenomics, spanning the field from discovery to clinical implementation. Seven papers focus on the discovery of novel variants and/or associations, three focus on technological developments, and five cover different aspects of the clinical translation of pharmacogenomics.

Cismaru et al. [1] performed a genome-wide association study (GWAS) to identify genetic variants implicated in metamizole-induced agranulocytosis (MIA) and neutropenia among three European populations. In their joint meta-analysis of MIA cases across all cohorts, two candidate loci on chromosome 9 were identified, rs55898176 (OR = 4.01, 95% CI: 2.41–6.68, *p* = 1.01 × 10^−7^) and rs4427239 (OR = 5.47, 95% CI: 2.81–10.65, *p* = 5.75 × 10^−7^). This was the first reported GWAS for MIA, which identified associations with biological plausibility that provide important insight into the mechanism underlying MIA.

In addition to the GWAS by Cismaru et al. [1], several candidate gene pharmacogenomic studies were also reported in this Special Issue. For example, de Carvalho et al. [2] employed a targeted genotyping panel of candidate genes and variants to study children from Brazil with acute lymphoblastic leukemia (ALL) undergoing chemotherapy with 6-mercaptopurine (6-MP) and methotrexate (MTX). An association was identified between the risk of death and the *TPMT* rs1142345 variant allele, which is found on both the TPMT*3A and *3C haplotypes. In addition to this Brazilian cohort study, 6-MP and MTX therapy was also studied by Kodidela et al. [3] in their childhood ALL cohort from South India. Targeted genotyping of 14 candidate pharmacogenomic variants identified an association between treatment-related toxicity and the *NUDT15* c.415C>T variant allele (HR: 3.04 (95% CI: 1.5–6.1); *p* = 0.007), which is found on both the NUDT15*2 and *3 haplotypes. These two studies support previous reports that characterized a strong association between *TPMT*/*NUDT15* and thiopurine toxicity, and expand the knowledgebase to now include these additional populations that previously have been underrepresented in the field of pharmacogenomics.

In addition to 6-MP treatment for pediatric ALL, thiopurine response was also studied by Harmond et al. [4]; however, their research was focused on azathiopurine (AZA) treatment of chronic inflammatory bowel disease (IBD), including Crohn’s disease and ulcerative colitis. The authors reviewed the role of AZA in IBD treatment, which included an overview of the previously characterized association with *TPMT* and the potential for genotype-guided clinical management. In addition, the authors included a case report that identified a novel loss of function *TPMT* variant allele (c.483_484del; p.Asp162Serfs*26) in a patient with ulcerative colitis and thiopurine sensitivity.

Bergmeijer et al. [5] reported their candidate gene study on platelet reactivity in clopidogrel-treated patients undergoing elective percutaneous coronary intervention (PCI), which included collaboration with the International Clopidogrel Pharmacogenomics Consortium (ICPC). Targeted genotyping of the CYP3A4*22 and *PPAR-α* (G209A and A208G) candidate genes identified a significant association between *PPAR-α* and reduced platelet reactivity (G209A AA: −24.6 PRU [−44.7, −4.6], *p* = 0.016; A208G GG: −24.6 PRU [−44.3, −4.8], *p* = 0.015). Importantly, their study design also accounted for the well-characterized association between *CYP2C19* variant alleles and on-treatment platelet reactivity, as well as other known clinical variables.

Gene expression pharmacogenomic research was also reported in this Special Issue, as detailed by Santos et al. [6]. The authors of this study sought to identify gene expression predictors of response to varenicline, which is indicated for smoking cessation. Gene expression analysis was performed using a custom qPCR array assay that included 17 candidate genes, and their cohort included 13 patients who were resistant to varenicline treatment and 14 patients who had a successful response with tobacco abstinence through four weeks of treatment. A significant decrease in *CHRNA7* gene expression was observed in the resistant group compared to baseline values (T2 fold change: 0.38, *p* = 0.007; T4 fold change: 0.67, *p* = 0.004, respectively). These exploratory results suggest that downregulation of CHRNA7, which encodes the nAChR alpha7 subunit, may be implicated in varenicline response among patients undergoing smoking cessation treatment. 

Díaz-Villamarín et al. [7] report a systematic review on the pharmacogenomics of antivascular endothelial growth factor (anti-VEGF) antibody therapy (e.g., ranibizumab, bevacizumab, and aflibercept) among patients with polypoidal choroidal vasculopathy (PCV) and age-related macular degeneration (AMD). Given that anti-VEGF treatments have shown variable efficacy, the authors performed a systematic review and meta-analysis that identified four variants (*CFH* p.I62V, *CFH* p.Y402H, *ARMS2* p.A69S, and *HTRA1* c.-625A/G) significantly related to response. Their results provide important directions for future studies to validate these findings in larger patient cohorts with PCV or AMD.

Genotyping technologies continue to develop at an unprecedented pace. In their review, Van der Lee et al. [8] provide a comprehensive overview of the potential advantages and limitations for application in the field of pharmacogenomics both for research as well as clinical practice. While panels of single nucleotide variants offer an attractive approach for clinical practice because of the short turnaround time, straightforward interpretation, and low cost, not all panels offer suitable coverage of pharmacogenomic regions and by design are limited by their ability to assess rare and structural variants. To this end, both long- and short-read sequencing offer more attractive solutions. The review also highlights several important challenges for the field including drug metabolizer phenotype inference of variants of unknown significance with computational tools and the complexity of the majority of pharmacogenes due to the occurrence of copy number variations, structural rearrangements, and repetitive regions. 

In an experimental paper by Botton et al. [9], the benefits of using high-resolution approaches such as long-read single molecule real-time (SMRT) sequencing are demonstrated. They show that this method outperforms high-depth short-read sequencing in the detection of complex variants and offers the advantage of phased resolution, which will enable more accurate predictions of drug metabolism phenotypes. 

Overkleeft et al. [10] highlight another technological challenge for making personalized medicine a reality: the need to have a complete overview of the genetic make-up of the patient, the medical family history, and the drug use and other health-related data at point-of-care that comply with privacy ethical standards. They describe their Personal Genetic Locker project, which aims to implement personal genomic data effectively in primary care.

After decades of research, the clinical translation of findings from pharmacogenomic studies to clinical practice remains challenging and there is substantial heterogeneity between countries, healthcare systems, and even between first/2nd/3rd line treatment setting with healthcare systems regarding the routine application of pharmacogenomic tests. Rollinson et al. [11] outline the potential utility of pharmacogenomics in primary care and discuss barriers for implementation that will be a major challenge over the next five years. They specifically discuss commonly used drugs in primary care with available pharmacogenomics guidelines. Interestingly, they highlight the importance of development of appropriate clinical decision support systems that facilitate the use of pharmacogenomic information at the point of prescribing, as also emphasized by Overkleeft et al. [10]. 

Taylor et al. [12] comprehensively review *CYP2D6*, one of the most important and widely investigated pharmacogenes that is involved in the metabolism of ~20% of commonly used drugs. They provide a nice background while also discussing some of the challenges attached to this complex gene, such as the occurrence of small insertions/deletions, larger structural variants, and hybridization issues with the neighboring non-functional *CYP2D7* pseudogene. The review ends with a discussion of novel approaches to *CYP2D6* phenotyping, ranging from saturation mutagenesis to long-read sequencing and deep learning approaches.

A major challenge for the field of pharmacogenomics is the paucity of sufficiently powered studies. These studies are challenging to recruit and many studies lack rich drug response phenotype data. Naik et al. [13] review the use of Digital Health solutions to overcome this problem and outline potential solutions to improve pharmacogenomic trial design and operation. These solutions are very attractive in that through the use of apps or wearable devices, they put the patient in a far more active role than is currently the case in trials.

In a review paper of the well-studied gene–drug interaction between *CYP3A5* and tacrolimus, Van Gelder et al. [14] shed light on how it is possible that the changes in tacrolimus exposure after switching to different modified release formulations are larger in patients who express the CYP3A5 enzyme (CYP3A5 *1/*3 or *1/*1) compared to nonexpressers (CYP3A5*3/*3). They hypothesize that this may be due to the fact that in the upper region of the small intestine CYP3A activity is higher, and that this expression of CYP3A decreases towards the more distal parts of the gut. 

De Jong et al. [15] discuss a fundamental challenge that is limiting our ability to predict drug metabolism based on genetic variation: the effect of inflammation on drug metabolism, a phenomenon that is often referred to as phenoconversion. To date, these effects are largely ignored; however, with the increasing clinical use of pharmacogenomics, they are rapidly gaining attention. In this review, the evidence from in-vitro models on the effect of inflammatory mediators on CYP450 activity is presented and mechanistic pathways via which inflammation in hepatocytes may modulate hepatic functions that are critical for drug metabolism are discussed.

In conclusion, this Special Issue of *Genes* entitled “*Pharmacogenomic Determinants of Interindividual Drug Response Variability: from Discovery to Implementation*” has enabled the publication of 15 international papers on pharmacogenomics, which include novel research and reviews that span the fields from discovery to clinical implementation. The Editors are grateful to all of the authors who performed and wrote these innovative articles, as well as the volunteer reviewers that independently adjudicated these manuscripts for consideration for publication.

## References

[1] Cismaru A.L., Rudin D., Ibanez L., Liakoni E., Bonadies N., Kreutz R., Carvajal A., Lucena M.I., Martin J., Sancho Ponce E. (2020). Genome-Wide Association Study of Metamizole-Induced Agranulocytosis in European Populations. Genes.

[2] de Carvalho C.D., Pereira Colares Leitao L., Mello Junior F.A.R., Vieira Wanderley A., Souza T.P., de Sa B.A.R., Cohen-Paes A., Rodrigues Fernandes M., Santos S., Salim Khayat A. (2020). Association between the TPMT*3C (rs1142345) Polymorphism and the Risk of Death in the Treatment of Acute Lymphoblastic Leukemia in Children from the Brazilian Amazon Region. Genes.

[3] Kodidela S., Dorababu P., Thakkar D.N., Dubashi B., Sundaram R., Muralidharan N., Nidanapu R.P., Aribandi A., Pradhan S.C., Uppugunduri C.R.S. (2020). Association of NUDT15*3 and FPGS 2572C>T Variants with the Risk of Early Hematologic Toxicity During 6-MP and Low-Dose Methotrexate-Based Maintenance Therapy in Indian Patients with Acute Lymphoblastic Leukemia. Genes.

[4] Harmand P.O., Solassol J. (2020). Thiopurine Drugs in the Treatment of Ulcerative Colitis: Identification of a Novel Deleterious Mutation in TPMT. Genes.

[5] Bergmeijer T.O., Yasmina A., Vos G.J.A., Janssen P.W.A., Hackeng C.M., Kelder J.C., Verma S.S., Ritchie M.D., Gong L., Klein T.E. (2020). Effect of CYP3A4*22 and PPAR-alpha Genetic Variants on Platelet Reactivity in Patients Treated with Clopidogrel and Lipid-Lowering Drugs Undergoing Elective Percutaneous Coronary Intervention. Genes.

[6] Santos J.R., Tomaz P.R.X., Scholz J.R., Gaya P.V., Abe T.O., Krieger J.E., Pereira A.C., Santos P. (2020). Profile of the Nicotinic Cholinergic Receptor Alpha 7 Subunit Gene Expression is Associated with Response to Varenicline Treatment. Genes.

[7] Diaz-Villamarin X., Blanquez-Martinez D., Pozo-Agundo A., Perez-Gutierrez A.M., Munoz-Avila J.I., Antunez-Rodriguez A., Fernandez-Gomez A.E., Garcia-Navas P., Martinez-Gonzalez L.J., Davila-Fajardo C.L. (2020). Genetic Variants Affecting Anti-VEGF Drug Response in Polypoidal Choroidal Vasculopathy Patients: A Systematic Review and Meta-Analysis. Genes.

[8] van der Lee M., Kriek M., Guchelaar H.J., Swen J.J. (2020). Technologies for Pharmacogenomics: A Review. Genes.

[9] Botton M.R., Yang Y., Scott E.R., Desnick R.J., Scott S.A. (2020). Phased Haplotype Resolution of the SLC6A4 Promoter Using Long-Read Single Molecule Real-Time (SMRT) Sequencing. Genes.

[10] Overkleeft R., Tommel J., Evers A.W.M., den Dunnen J.T., Roos M., Hoefmans M.J., Schrader W.E., Swen J.J., Numans M.E., Houwink E.J.F. (2020). Using Personal Genomic Data within Primary Care: A Bioinformatics Approach to Pharmacogenomics. Genes.

[11] Rollinson V., Turner R., Pirmohamed M. (2020). Pharmacogenomics for Primary Care: An Overview. Genes.

[12] Taylor C., Crosby I., Yip V., Maguire P., Pirmohamed M., Turner R.M. (2020). A Review of the Important Role of CYP2D6 in Pharmacogenomics. Genes.

[13] Naik H., Palaniappan L., Ashley E.A., Scott S.A. (2020). Digital Health Applications for Pharmacogenetic Clinical Trials. Genes.

[14] Gelder T.V., Etsouli O., Moes D.J., Swen J.J. (2020). Comparison of the Impact of Pharmacogenetic Variability on the PK of Slow Release and Immediate Release Tacrolimus Formulations. Genes.

[15] de Jong L.M., Jiskoot W., Swen J.J., Manson M.L. (2020). Distinct Effects of Inflammation on Cytochrome P450 Regulation and Drug Metabolism: Lessons from Experimental Models and a Potential Role for Pharmacogenetics. Genes.

